# Swimming with robots: investigating fish locomotion, sensing, and schooling behavior with robotic swimmers

**DOI:** 10.1038/s41467-026-72478-6

**Published:** 2026-05-02

**Authors:** Auke Ijspeert, Francesco Mondada, Emily Standen, Guy Theraulaz

**Affiliations:** 1 EPFL-BioRobotics Laboratory, ME D1 1226, Station 9, Lausanne, Switzerland; 2 EPFL-MOBOTS, MEB3426, Station 9, Lausanne, Switzerland; 3 University of Ottawa, Comparative and Evolutionary Biomechanics Laboratory, 160 Gendron Hall, 30 Marie Curie Pvt., Ottawa, ON Canada; 4 Centre de Recherches sur la Cognition Animale, Centre de Biologie Intégrative, CNRS, Université de Toulouse, Toulouse, France

**Keywords:** Mechanical engineering, Biomedical engineering, Social sciences

## Abstract

Fish display remarkable locomotor and social abilities, from efficient swimming to coordinated schooling, that have inspired the design of various robotic fish. While robotics has largely benefited from biology, fish-like robots are increasingly used as scientific tools to investigate fundamental questions in biomechanics, sensorimotor control, and collective behavior. This paper reviews how robotic models have been employed to study the neuromechanical basis of swimming, the role of sensory feedback in locomotion, and the mechanisms underlying social interactions in schools. We list open questions in biology and show that robotic approaches provide unique advantages to address them: they enable repeatable experiments, systematic variation of body and control parameters, and direct measurement of otherwise inaccessible quantities such as internal forces or energy use. A literature analysis reveals, however, that only a minority of robot-fish studies contribute to biological understanding, with most focusing on engineering design. Among biology-oriented studies, closed-loop robotic systems—capable of real-time adaptation—remain underrepresented but are essential for probing sensorimotor and social feedback mechanisms. We conclude by outlining future directions combining robotics, simulations, and emerging experimental technologies to unravel the multi-scale feedback loops that shape fish locomotion and schooling.

## Abstract (web summary)

Robotic fish are shifting from engineering curiosities to experimental partners in biology. This Perspective highlights how adaptive, closed-loop robots enable controlled tests of neuromechanical, sensorimotor and social feedback underlying fish behavior.

## Introduction

When observing single and groups of fish swimming, one is often impressed by the elegance and agility of their swimming. Fish exhibit a large richness of behaviors that switch between slow and smooth movements, agile navigation around obstacles, rapid bursts of acceleration, as well as strikingly coordinated movements when swimming in a school. These remarkable behaviors have attracted the attention from scientists in various research fields such as hydrodynamics, biomechanics, neuroscience, ethology, and evolutionary biology^1–3^. Some fish, like the zebrafish, have become important model animals in multiple fields of biology like neuroscience, developmental biology, genomics, and pharmacology^4–6^.

**Fig. 1:**
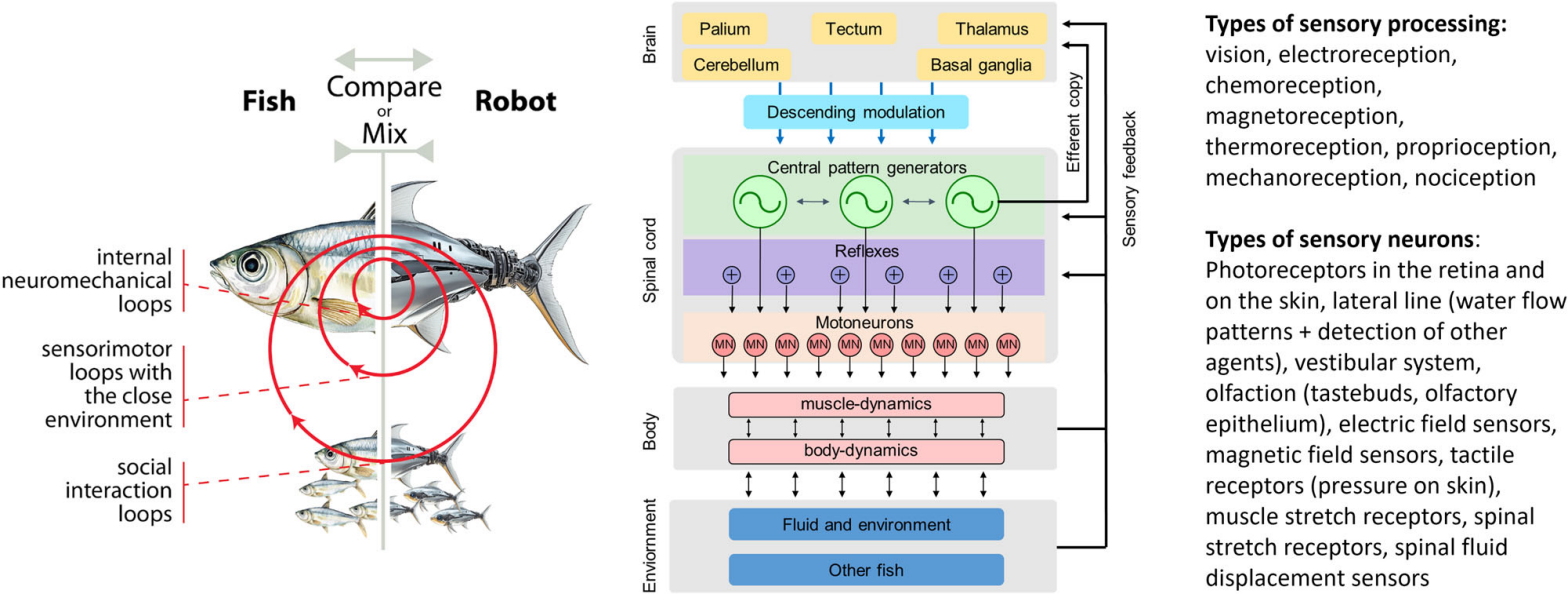
Multi-nested feedback loops in swimming fishes. (Left) Different feedback loops in fish that can be investigated with robots. (Right) Typical neural architecture in a fish, with different types of supraspinal and spinal sensory processing. Locomotion is coordinated in the spinal cord based on central pattern generators (CPGs, neural networks that produce rhythmic patterns) and reflex loops. The brain receives sensory feedback signals from the body, and an efferent copy of CPG activity, as well as sensory signals from the eyes and from the vestibular system. The brain is made of different centers, including the pallium (equivalent to the cerebral cortex in mammals) that produces motor plans, the tectum that acts as the primary visual center, the cerebellum that is involved in coordination and learning, the thalamus that process multiple sensory modalities, and the basal ganglia that is involved in action selection^12^. These brain centers (except for the thalamus) then activate and modulate locomotor patterns through a finite set of descending modulation pathways that reach the spinal cord through the brain stem. Multiple types of sensory processing are taking place throughout the nervous system.

Fish are also interesting for engineers to replicate their agile and energy-efficient behaviors in robots or to improve the agility and efficiency of marine vehicles^7–10^. Over the last 30 years, many studies have developed fish-like robots, and over 2500 articles have been published on the topic. Among these, a subset of studies has developed robots (or robotic tools) specifically to address biological questions. In particular, robots can be used to study the neuromechanics of fish swimming, i.e., the coupling of the body mechanics (the musculoskeletal system interacting with water) and the nervous system when fish swim and navigate in their environments. This leads to multi-nested feedback loops (Fig. 1). The first feedback loops are internal neuromechanical loops. These loops depend on the physical interactions of the body with the water, where body and fin muscle-induced movements create reaction forces through contact with the water, thus allowing the body to move and turn. The muscle activity is produced by neural networks in the spinal cord called central pattern generators (CPGs) that produce rhythmic neural activity^11–13^. These CPGs receive inputs from proprioceptive (i.e., internal to the body) sensors that measure deformations and internal forces across the body, such as to keep CPGs coordinated with the actual movements of the body^14^.

The second feedback loops are based on proximity exteroceptive (i.e., at the interface or external to the body) sensors that can feel the local pressure and flow of the water (through tactile sensory neurons and the lateral line)^15,16^. These feedback loops modulate CPG activity and allow the fish to react to changes in the water flow, to optimize its swimming (e.g., in terms of speed or efficiency), and to maintain its orientation and position. Some fish additionally use electric field sensors or other active mechanisms to adapt to local obstacles and to other life forms in the vicinity^17^.

A third feedback loop is created through the social interactions with other fish. Many fish coordinate their swimming with conspecific fish through shoaling and schooling behaviors^18,19^. Several sensory modalities are involved in these processes, including vision, which provides information about neighbor position and orientation; the lateral line system, which detects local flow and hydrodynamic disturbances; and proprioceptive feedback, which contributes to locomotor control and body coordination^14,20^. Beyond social coordination, fish exhibit a wide range of behaviors essential for survival, including predator avoidance, navigation, feeding, hunting, and mating, all of which rely on the integration of multiple sensory channels and adaptive decision-making^5,18^.

In the next sections, we will first discuss why robots can be useful to study fish and provide a literature review of related work. We will then discuss more in depth how robotics has been used to study the different feedback loops discussed above, starting from neuromechanical loops involved in swimming up to sensorimotor principles of fish schooling. For each type of loop, we will discuss open questions in biology and present some examples of how they have been addressed using robots. Compared to the vast literature and reviews of fish-like robots, where biology has been taken as inspiration for engineers to build innovative and efficient fish-like robots^7–10,21,22^, our focus will mainly be on the use of robots to address scientific questions in biology. We also focus particularly on studies that have investigated sensorimotor control principles from single to multiple fishes.

## Why use robots to study fish?

Since experimental setups and genetic tools are making good progress, one might ask what is the usefulness of using robots compared to performing experiments only with real animals. The use of robots to investigate animal behaviors is indeed a growing field, see^23–25^ for reviews. The main reason is that robots offer the opportunity to collect data otherwise inaccessible, with a methodology and regularity otherwise unachievable. For instance, robots offer the possibility to systematically change physical properties of the body (size, weight, geometry, stiffness, …) and investigate their effects on locomotion and influence on other fish. They also allow one to record internal quantities that are difficult to measure in animals (e.g., internal torques, internal stretch, power consumption, and cost of transport), and therefore provide proxy estimates of these quantities in animals. Robots can also be programmed to test behaviors that are not observed in animals, for instance, different swimming gaits, as well as different behaviors within a school. This is interesting to investigate why fish exhibit particular behaviors and not others, and to reverse engineer possible criteria that are optimized when selecting behaviors. Also, unlike experiments with animals, robots can be disrupted or lesioned without ethical restrictions. Finally, robots can be particularly useful to demonstrate that a particular sensorimotor mechanism is sufficient (as opposed to necessary) to reproduce a particular behavior. Indeed, biological experiments can show the necessity of a mechanism through ablation studies, but demonstrating sufficiency is much more difficult because of the experimental difficulty of deactivating all possible sensorimotor loops except one. For these reasons, the biological questions that can be effectively studied with robotic fish fall into two categories; mechanistic questions and behavioral questions. Mechanistic questions are those that ask about the potential function of specific characteristics we see in biology; characteristics that we can build into our robots and then manipulate and test repeatedly in a controlled way relative to other built-in traits (in the spirit of Richard Feynman’s famous quote: “What I cannot create, I do not understand”). Mechanistic questions can also explain physical interactions between animals and their environment, such as hydrodynamic efficiency and performance. Behavioral questions are those that require close observation of living animals, which can be answered with robots that can exist in close proximity without disrupting natural animal behavior or can actually be used to purposefully alter fish behavior in a repeatable way.

The challenges of designing and constructing robots for scientific studies should, however, not be underestimated. As discussed next, simple physical (i.e., non-actuated) models or numerical models might sometimes be better suited. Indeed, there are many practical (e.g., waterproofing) and technological challenges that need to be solved for making robots that properly approximate animal bodies, for instance, in terms of (high) numbers of degrees of freedom and viscoelastic properties. Replicating small fishes is particularly difficult because the power density of actuators does not scale well with smaller volume and because of the difficulty of miniaturizing electronics. Several sensor modalities, such as waterproof tactile skins and lateral lines, are also difficult to replicate (although there is interesting progress in that direction, see^26,27^). Finally, for studying social interactions between robotic and real fish, other aspects such as the appearance, the behavior, as well as possibly the chemical and electrical signatures should also be considered.

## Why robots and not numerical simulations?

Numerical simulations of fish (including body models, control circuits, and fluid dynamics) can be useful to complement animal and robot experiments^28–33^). Compared to physical robots or simpler devices, the advantages of numerical simulations are that they offer more freedom to change body properties, they can better match fish biomechanics (e.g., the simulation of muscles and tendons), they can more easily simulate all kinds of sensors (because of the access to all internal states of the simulation), and they are well-suited for optimization/learning. Real devices, on the other hand, benefit from real physics and do not suffer from approximate fluid dynamics simulation (or any other numerical artifact). They also offer more realistic visual inputs (e.g., in terms of complex sceneries, turbidity, etc.). Finally, real robots are essential for physically and visually influencing real fish group behaviors. As we will see next, several studies combine robot and simulation experiments, typically with robot experiments validating that simulation experiments properly transfer to the real world^34–36^.

## Literature review of studying fish swimming using robots

**Fig. 2:**
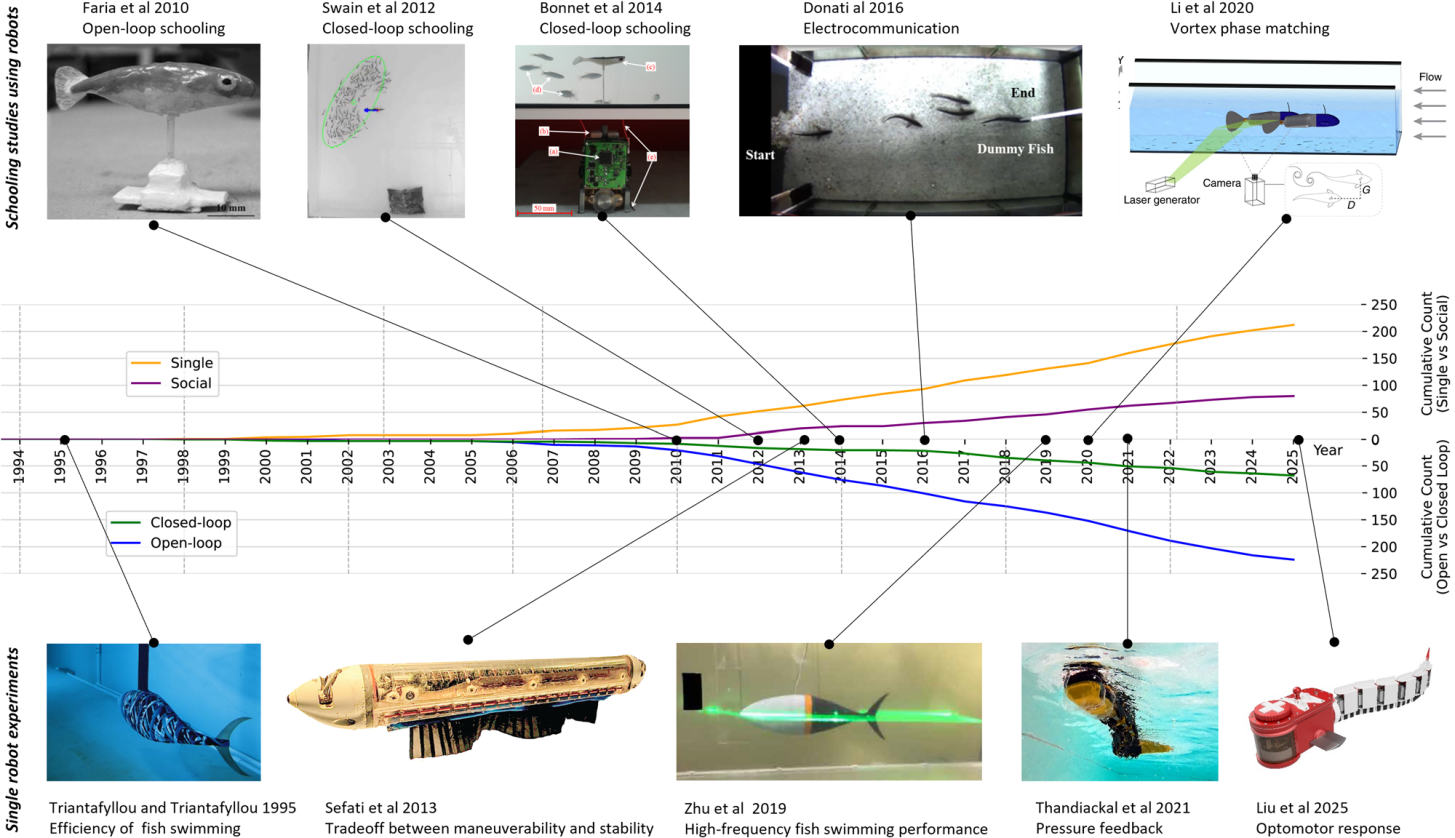
Timeline of swimming robots used to address biological questions. The top row shows examples of robots used to study social and schooling behaviors. The second row shows the cumulative time evolution of articles that use robots to investigate (single) fish swimming and (social) fish group behavior. It also distinguishes articles that use robots in open-loop control versus closed-loop control. The bottom row shows examples of single fish robots used to investigate questions about the fluid dynamics and sensorimotor control of fish swimming. Images adapted with permissions from references (Springer-Nature)^112^, (IEEE, CCC license)^142^, (IEEE, CCC license)^116^, (IOP Publishing, CCC license)^100^, (Springer-Nature)^79^, (Springer-Nature)^44^, (PNAS)^49^, (AAAS, CCC license)^47^, (AAAS, CCC license)^34^, (AAAS, CCC license)^36^.

There are thousands of papers investigating the synergies between the understanding of fish biology and the design of robotic fish. But looking into more detail (Box 1), the synergies seem to go mainly one way: from biology to robotics. Bio-inspired robotic fish design is explored in close to 2600 papers in the literature (from Web of Science, method in Appendix), while the papers where robotics serves a better understanding of fish biology are represented in less than 300 papers (Fig. 2).

Among these 292 papers having a biological contribution based on a robot, we looked at whether the robotic fish was acting in open or closed loop, namely whether the robot controller was implementing fixed swimming patterns open loop, or using (on-board or off-board) sensors to adapt to the environment and/or to other fish in a real-time closed loop. This classification resulted in 68 closed-loop papers and 224 open-loop papers, which unveils that only a minority (23%) of the biology-oriented papers implemented closed-loop experimentation. Most of those 292 papers addressed body and movement mechanics (179), and in this class, most of the papers were open-loop tests (15% were closed-loop). Most closed-loop papers deal with control (39) or social behaviors (32). Even in papers presenting robot-fish interactions (103), only 31% of the papers investigate closed-loop interactions.

Looking at the time evolution (Fig. 2), it is interesting to see two waves of papers presenting closed-loop interactions, one between 2006 and 2014, where most of the papers are investigating the mechanical properties of swimming, and a second wave of papers, from 2016 to today, has a much higher percentage of papers dealing with fish social interactions explored with the use of robots. We see here a shift from studies of the inner feedback loops toward outer feedback loops.

Robotic models have contributed many unique biological insights in three main areas; swimming mechanics, sensory control and collective behavior. For example, robots have been used to demonstrate that (i) peripheral sensory feedback alone can, in principle, generate traveling body waves, (ii) local hydrodynamic and visual cues are sufficient to drive social coordination, and (iii) tunable body stiffness optimizes swimming efficiency. These and other findings are described below.

Box 1: Method and extended results of the literature review We used “web of science” to find all papers where the terms “robot*” and “fish”, or terms of some specific fish species such as lamprey, zebrafish, carp, guppy, golden shiner, hemigrammus, tuna, eel, trout, boxfish, mackerel, manta, knifefish, salmon, shark, catfish, or Mosquitofish were used anywhere in the paper summary, including the title, abstract and keywords. We removed search results such as news items, editorial material, corrections and meeting abstracts. This search resulted in 5250 papers. We then classified the results based on their abstract and title, into papers with an emphasis on fish biology using a robot as a tool, and those with an emphasis on robotic fish design. A group of papers were found matching the two categories and making a stronger link between the two fields. To make this sorting, we used ChatGPT 4o, prompting the chatbot, for each title and abstract, to first say if this was a biologically-oriented paper using robots and then, in a second prompt for the same paper, to say if the paper was only engineering-oriented. This two-prompts methodology allows the chatbot to have a single clear context. Preliminary tests asking the chatbot with a single prompt to classify the paper into one of the two classes have shown to be less precise. The precise text of the two prompts were fine-tuned in several rounds based on a subsample of papers. To validate the final version of the prompts and the resulting classification, 50 randomly selected papers were classified by an expert and compared with the classification made by ChatGPT, resulting in 94% of agreement between the expert and the chatbot, which is a high rate of agreement and validated the classification. To further ensure a quality of classification, the final decision on the classification was made based on the majority of three chatbot feedbacks.

As output of this classification, we have 2698 papers that have been classified in one or both categories, the remaining papers dealing with other aspects, from fishing to entertainment. Among these 2698 papers, 292 papers are matching the biological category, 2586 are matching the robotics category, and among them, 180 papers are matching both categories. We observe that papers contributing to biological questions are a small minority of this literature (12%). Among the 292 papers having a biological contribution based on a robot, we tested, using the same approach, if the robotic fish was acting in open or closed loop. This classification was much harder, and in this case, we asked the chatbot to justify each choice, which again has shown to provide better results, despite using more output tokens. The chatbot classified 232 papers, the others being considered too difficult to classify. To check the classification, 30 random papers ( > 20%) were classified by an expert, and the final agreement with the chatbot resulted to be at 93%, validating the classification. The remaining unclassified papers were classified by hand by an expert, who accessed not only the title and abstract, but also the article content. Papers containing both approaches, for instance review papers, were classified under the closed loop. This resulted in 68 closed-loop papers and 224 open-loop papers. This shows that only 23% of the papers with a biological interest present closed-loop mechanisms implemented in the robot. Most of the papers in these 292 were papers addressing body and movement mechanics (179), and in this class, most of the papers were open-loop tests (15% were closed-loop). Most closed-loop papers deal with control (39) or social behaviors (32). Even in papers presenting robot-fish interactions (103), only 31% of the papers are on closed-loop interactions. All these classifications have been validated by experts.

## Investigating the mechanics of fish swimming using robots

Fish exhibit remarkable swimming skills in terms of speed, efficiency, and agility. For instance, blue marlins and sailfish have been recorded pulling a line out of a fishing reel at speeds over 100 km/h [BBC One Plant Earth Episode: Deep Ocean]. Eels and salmon can swim thousands of kms without food^37,38^. And in fresh and salt water, fish burrow in sediment, swim in turbulent flow and maneuver between complex habitat structures with ease.

Fascination around how fish swim is not new. Aristotle in his work ‘On the parts of animals’ was the first to suggest that the body and tail fin were essential for producing thrust in locomotion. Classification and description of fish locomotion styles were first canalized by Breeder in 1926 and have been categorically defined into four types of body caudal fin swimming as well as different forms of paired and median fin swimming ever since^39^. Since then, work has shown that body caudal fin swimming categorization has less to do with differences in kinematics and more to do with morphological differences between species, and so caution should be used when considering the significance of different swimming ‘types’^40–42^. Regardless of the classification of fish swimming, it is interesting to note the effect that different swimming behaviors have on sensory perception and resultant neuro-control strategies^17,43^. For example, burst-and-coast swimming may provide for periods of high-resolution sensory perception during gliding with noisy perception during bursts. Pectoral fin swimming with the body straight may allow for heightened sensory resolution, while body caudal swimming may require more filtering of body-induced signaling when determining important external sensory cues. Also, some fish appear to choose swimming gaits that are suboptimal in terms of energy efficiency in order to improve their perception, as shown in a computational model of the weakly electric fish that swims with a tilted angle^43^.

The diversity of fish body form and function is remarkable, with over 35,000 species inhabiting every possible aquatic niche on the planet and providing a rich potential for understanding how the neuromotor systems work in different mechanical and sensory-rich environments. However, the complexity and integration of different neurocomponents used for swimming in biological systems make it difficult to understand how each contributes to effective locomotion in living animals. Robots and their capacity to be specifically manipulated provide an excellent model for deconstructing the critical control and fluid dynamics components of fish swimming.

Since the pioneering work on the Tuna robot of MIT^44^, a series of robots have been designed to address scientific questions related to the mechanics of fish swimming. These robots come in different implementations: (i) Actuated robotic fins attached to a fixed structure usually in a flow tank^44,45^; (ii) self propelled robots attached to a low-friction rail^8,46–48^; or (iii) freely moving fish-like robots^24,34,49–51^, see some examples in Fig. 2. Initially most robots were designed with hard components and traditional electromagnetic motors, but more recently soft actuators (e.g. pneumatic, hydraulic, dielectric or others) and soft components are increasingly used^45,50,52–54^. Note that the robotic projects we will discuss in this article are overlapping with studies that use physical models (passive or remotely actuated) to investigate fish swimming. Such physical models have been useful to study functional properties of shark skin^55^ and the fluid dynamics of schooling behavior^56,57^, for instance. We see physical and robotic systems as part of the same spectrum of physical devices, from passive to open-loop to closed-loop behavior, with increasingly complex influences and responses. Because biological systems are not passive, we will mainly focus on studies that use robots (with on-board or off-board actuation) and that implement closed-loop control, but we will also present projects that use simpler robotic devices.

Using robots, one can address multiple questions stemming from biologists: (i) How do fish morphology, movement and body and fin stiffness relate to swimming performance? (ii) How are tradeoffs like speed, efficiency, stability and maneuverability satisfied? (iii) How and why do animals switch between swimming modes, and how is it related to morphology and environment? (iv) What are the underlying control mechanisms that allow fish to be so flexible and variable in their locomotion? And (v), how have evolutionary constraints shaped the fish of the past and of today? The capacity of robots to use optimization to test relationships between morphology, movement, and performance makes them a powerful tool to reflect on why we see particular patterns in behavior and morphology in the natural world.

When observations of the biological world cannot be explained by application of established physical or biochemical laws, robots can provide a method of testing proposed hypotheses. For example, Gray’s Paradox suggests that the muscle capacity of a dolphin is much less than that required to overcome the drag experienced at observed maximum swimming speeds^58^. Over the years, people have tried to explain the paradox through a better understanding of how dolphins may be reducing hydrodynamic drag^59–61^, or by suggesting that using better muscle power calculations could improve estimates of dolphin maximum performance^62,63^. Interestingly, one of the main purposes for designing the seminal MIT Tuna robot was to address Gray’s paradox using experiments with a robot^44^. Experiments showed that “the power required to propel an actively swimming, streamlined, fish-like body is significantly smaller than the power needed to tow the body straight and rigid at the same speed”^64^.

In addition to shape changes, it is generally believed that the stiffness of the swimming body as it moves through the fluid also has a large impact on the efficiency of thrust generation^58^; essentially, the rigidity of structures influences their capacity to produce hydrodynamic thrust^65^. Multi-joint and tunable robotic systems with variable and dynamic stiffness show that the timing of body stiffening is of critical importance when considering efficiency and speed^66^ and can even result in the emergence of different gaits^67^. Robotic models also show that fin stiffness is important in thrust production, with stiffer caudal fins producing more thrust^48,68^. Biological systems such as the Bluegill sunfish also appear to tune their fins, increasing tail stiffness with speed^69,70^. The inherent properties of biological muscle are also capable of remarkable changes in stiffness, suggesting fishes use these dynamic stiffening strategies to increase speed, efficiency and to change gait. Physical models of fish scales and skin have shown these tissues are also important factors in determining steady swimming, providing elastic energy and stiffness differently to different regions of the body^71^.

Robots can also be used to investigate different trade-offs, for instance between stability and maneuverability^49^ and between speed and energy efficiency in swimming^72–74^. Unlike biological organisms, robots can be programmed to generate a large diversity of swimming gaits and can record power consumption during swimming. For example, a study using an elongate robot revealed that the speed and energy efficiency of swimming depend strongly on the amplitude and phase lag of body undulations, with different optimal gaits arising for each optimized criteria^72^. Compared to biological data, the study suggested that live eels tend to choose efficiency over speed. In another example, Zhu et al^47^ use the Tunabot to understand subtle components of tail fin curvature and amplitude that result in a more effective angle of attack of the tail, explaining how scombrid fishes increase their thrust production so effectively. Similar use of computational fluid dynamics models has been used to determine potential hydrodynamically ‘beneficial’ behaviors which can then guide robots to help explore parameter space in the real world. Robotic models used with flow visualization techniques help validate CFD models and understand the benefits animals are gaining from particular behavioral patterns they use^75–78^. As we will see later, many hydrodynamic experiments are focused on schooling fishes and the benefits they gain from moving in a structure together. For example, experimental foils in staggered and side-by-side formation show that the trailing vortex creates a hydrodynamic force that stabilizes the school formation. Using constrained or free swimming foils, robotic components show fish gain an efficiency, thrust and speed advantage when swimming together suggesting that a fluid-mediated equilibria may be adding performance and stability to schooling fish^79,80^, see the section *Unlocking Collective Animal Behavior Through Fish-Robot Interactions* for discussions.

As technology advances, and robots become more instrumented and autonomous, they are able to test hypotheses on how fish sense their environments. For example^81^, used a simple physical model to show that trout use side-to-side head oscillation to reduce sensory interference from body motion. In addition, efficient propulsion is achieved by specific yaw and heave of the head coupled with the correct phase angle. Exploration of the sensory capacity of fins has also been aided by robotic approaches^82^ where robotic fins with sensory membranes attempt to replicate sensory feedback observed in fishes.

### Future research directions in investigating the mechanics of fish swimming

The scientific questions listed above have only been partially addressed and usually under steady swimming conditions. Biological experiments on live animals in both steady and unsteady conditions continue to put forward hypotheses that can be tested using robotic models. As our robotic models become more smoothly actuated, with more sensing capacity, we will be able to switch from open-loop to closed-loop systems that can answer complex questions surrounding fish agility, in particular, how fish perform three-dimensional maneuvers, as well as rapid accelerations and decelerations. The capacity to explore the diversity of fish morphology and modes of swimming with responsive and self-correcting robotic systems will provide great insight into what drives evolutionary diversification in terms of fluid dynamics and control.

## Reverse-engineering the sensorimotor circuits of swimming

Thanks to extensive studies of the neural circuits in animals like lamprey (a primitive eel-like fish)^11,83^ and zebrafish^13,14,84^, we now have a good understanding of the general organization of the neural architecture underlying swimming in fish (Fig. 1). The swimming circuits are based on central pattern generator (CPG) networks in the spinal cord. CPGs are distributed networks of local oscillatory circuits that are coupled together through neural projections. In lamprey, the swimming CPG is composed of a chain of local oscillators distributed along the spinal cord^11,83^. Zebrafish have a similar architecture, with additional specific oscillators generating distinct oscillatory frequencies that can be recruited for different swimming speeds^13^. CPGs can be viewed as feedforward controllers for swimming speed control^85^ that transform input signals coming from higher brain centers into oscillatory patterns necessary for swimming, with typically higher input signals leading to higher frequencies (faster swimming). They can also be used for turning maneuvers by differentially driving the left and right sides of the circuit^11,83^. Many swimming robots are now controlled with controllers that replicate CPGs as nonlinear oscillators or neural networks^34,86–90^. Most of these neuro-controllers tend to be implemented in software on on-board micro-controllers or computers, but several projects also explore how to implement them in dedicated neuromorphic hardware^86,91–95^.

Fish adapt their locomotion to the environment using feedback loops from different types of sensors (Fig. 1): e.g., stretch sensors (in the spinal cord and in muscles), tactile mechanoreceptors, the lateral line system (which measures changes of flow and pressure around the fish), the vestibular system (which provides information about head motion and spatial orientation), and vision^14–16,96–99^. Some fish also have an electric sense that provides information about local obstacles and other living beings^17,100^. All these sensor modalities will affect CPG activity either through direct feedback to the spinal cord or through descending modulation from higher centers. These sensorimotor loops contribute to robustness against perturbations, and, as we will see next, against injuries. They also help in coordinating different parts of the body and, as we will see in the next section, in synchronizing the frequencies of different fish swimming in a school.

Whereas the general architecture of the swimming circuits is known, many questions remain both about the detailed organization and the function of the underlying sensorimotor control circuits, and how they deal with the complexity of the animal body and its environment. These questions include: (i) How are multiple sensor modalities combined into functional sensorimotor responses? (ii) How do they balance feedforward vs feedback control? (iii) What are the respective gains of different sensorimotor loops, and are some sensor modalities more important than others? (iv) Do these gains change over time, depending on the motor behavior and the environment? (If yes, on which time scale?) (v) How can sensory feedback contribute to providing robustness against lesions?

By systematically implementing and testing different feedback loops, robots and simulations can be ideal tools to address these questions. As mentioned earlier, robots can be particularly useful to demonstrate that a particular sensorimotor mechanism is sufficient (as opposed to necessary) to reproduce a particular behavior. They can also be useful to reverse engineer sensory gains through systematic tests.

The role of proprioception, in particular stretch feedback, in handling perturbations has been studied using neuromechanical simulations of lamprey and salamander swimming^29,101,102^. These simulations have shown that feedback from stretch sensors (called edge cells) within the spinal cord itself can serve as a local stiffening mechanism that prevents excessive bending and can, for instance, help the lamprey to cross a speed barrier (i.e., a perturbation in the flow with locally higher velocities). Such feedback mechanisms could potentially also be used for tuning the body stiffness, such as to obtain energy-optimal swimming at different speeds^103^, but this remains to be tested with real animals. Neuromechanical simulations have also shown that proprioception could play a role in recovery of spinal cord lesions in the lamprey, in particular, when the gains of the sensory feedback loops are increased after the simulated injury^33^.

The role of exteroception has been investigated in different studies with real robots. For instance, using an anguilliform robot equipped with load-cells (Fig. 2), Thandiackal and colleagues have shown that tactile pressure feedback can serve as a synchronization mechanism for undulatory swimming^34^. Remarkably, coordinated swimming could be obtained without direct couplings between oscillators of the CPG and rely on the local sensory feedback as synchronization signals. This suggests that multiple mechanisms for wave generation could co-exist based both on central and peripheral mechanisms. The study also showed that controllers that combine coupling and feedback are more robust against simulated injuries (random deactivation of couplings, feedback signals, and local oscillators) compared to any of these mechanisms alone.

In a recent follow-up study, the combination of proprioception (stretch feedback) and exteroception (tactile pressure feedback) was studied with the same robot^35^. It was shown that stretch and pressure feedback work well together to generate swimming patterns. Interestingly, these feedback loops can also contribute to dry ground locomotion, and the robot was able to crawl forward by pushing against pegs, similarly to eels that are capable of moving on the ground between rivers. The simulated circuits could furthermore replicate the remarkable ability of eels to keep swimming shortly after a full spinal cord transection, a feat that is quite unique among vertebrate animals (most would suffer from paralysis or severe disruption). It was found that sensory feedback and the ability of oscillators to spontaneously oscillate are likely explanations for keeping the neural oscillators active and coordinated below the transection.

Robots have also been used to study how fish perform rheotaxis, i.e., the ability to keep a particular position and orientation in a flow, typically for station holding with the head oriented against the flow^20,104^. This behavior involves multiple sensor modalities, including the lateral line, vision, tactile pressure sensors, and the vestibular system^104^. As reviewed in^105^, different types of sensor technologies have been developed for replicating the lateral line in robots. Such flow sensors have been used with closed-loop control to demonstrate that two^106^ or more^26,27^ pressure sensors could be used for station keeping in different conditions. When the conditions are simple (uniform flow), simple control loops like those of Braitenberg vehicles^107^ are sufficient for station keeping^106^, while in more complex flow conditions, more sophisticated controllers based on recursive Bayesian filters are needed^27^.

Vision is also an important sense for many fishes. Using a robot and detailed simulations of the visuomotor control circuits identified in larval zebrafish, Liu and colleagues^36^ have investigated the zebrafish optomotor response, a visual stabilization behavior. The robot and simulation could replicate the typical behaviors of the larvae when presented different types of visual stimuli. The study even helped to identify new types of neurons involved in the process. The study also demonstrated that the visuomotor circuits could contribute to rheotaxis when the robot was tested in a real river with complex fluid dynamics and visual environments.

### Future research directions in investigating the sensorimotor circuits of swimming

There remain many interesting open questions related to the sensorimotor circuits of swimming. A first question is related to how different sensor modalities are combined and integrated into the various motor behaviors exhibited by fish. So far, most of the robotic studies have investigated one sensor modality at a time, whereas many motor behaviors, for instance rheotaxis and adaptation to perturbations, are influenced by multiple sensor modalities. Robotic studies are ideally placed to investigate how different sensor modalities are combined. By implementing different potential sensorimotor loops and systematically testing them, one could investigate how sensory information is combined (for instance, by weighted linear combinations or more complicated non-linear operations), and reverse-engineer the respective gains for different sensor modalities (possibly identifying whether one sensor modality has a stronger influence than others).

Similarly, much work remains to be done both in terms of neuroscience and robotics to decipher the whole control architecture of fish and how it implements all the different behaviors necessary for surviving, from obstacle avoidance and navigation, to hunting, feeding, escaping and mating. This will involve investigating and modeling how fish perform action selection, as well as their ability to adapt and to learn, as investigated in various fish studies^108,109^.

Finally, it will also be interesting to perform more comparative studies, both with a larger variety of real fish and robots, to investigate how behaviors and sensorimotor circuits are similar and different between fish species. Indeed, fish exhibit a large diversity of morphologies, modes of swimming, as well as environments (for instance, rivers, lakes, coral reefs or deep sea), and it is not yet clear to which extent they share similar sensorimotor circuits or not.

## Deciphering collective animal behavior through fish-robot interactions

Understanding the mechanisms behind collective animal behavior has long been a challenge for scientists, particularly in schooling fish where movement coordination and social interactions take place in a dynamic three-dimensional environment^19,110^. This challenge translates into a series of key biological questions: (i) what are the proximate interaction rules that individual fish follow, and which sensory channels (such as vision or the lateral line) are actually used to perceive neighbors and the surrounding environment? (ii) Which neighbors exert the strongest influence on a focal fish, and are interactions best described by metric, topological, or more dynamic networks? (iii) How do these simple local behavioral rules scale up to generate collective patterns such as polarization, milling, or coordinated escape waves, and how robust are these emergent structures to noise, density changes, or ecological perturbations? (iv) Why has schooling evolved and persisted across taxa, and what adaptive benefits does it confer in terms of predator avoidance through dilution and confusion effects, information transfer or hydrodynamic efficiency? (v) How do endogenous factors such as hunger, stress, or habituation modulate behavioral parameters and thereby alter group-level dynamics? (vi) Finally, how can controlled experiments combined with mechanistic, biologically grounded models provide the bridge between individual-level processes and the emergent properties of fish schools? Traditional behavioral studies often rely on observing live fish in controlled conditions, but these methods present limitations in manipulating and isolating key variables^111^.

The integration of biomimetic robotic fish into behavioral research has transformed the study of collective behavior, offering a controlled and repeatable method to analyze how fish respond to social stimuli^112–115^. Advances in robotics have made it possible to explore previously inaccessible aspects of fish behavior, including the role of hydrodynamic interactions, sensory perception, predator-prey interactions, and collective decision-making, ultimately contributing to a better understanding of social cognition in these aquatic species^79,116–120^.

The challenge lies in designing robotic fish that are not only visually and kinematically convincing to real fish but also capable of integrating into natural shoals while minimizing perturbations to group dynamics. By refining these robotic models and improving their ability to interact with live fish, researchers aim to bridge the “biomimicry gap” (*i.e*., the difference between artificial agents and their biological counterparts) while gaining unprecedented insight into how fish synchronize their swimming^121–123^. However, such metrics do not necessarily guarantee that all interaction rules or higher-order statistical properties remain unchanged, and subtle behavioral adaptations may still occur. More broadly, the use of robotic fish to test biological hypotheses requires explicit validation criteria. A robot should ideally reproduce not only superficial appearance or locomotion patterns, but also the behavioral responses it elicits in live animals and the statistical structure of their interactions. In this sense, validation can be framed in a “Turing-style” perspective, where the relevant criterion is whether the responses of real fish to a robotic stimulus are indistinguishable, according to defined behavioral metrics, from those observed in interactions with real conspecifics. Similar operational validation approaches have been advocated in neuroscience and artificial intelligence research^124^ and are increasingly implemented in closed-loop biohybrid experiments and behavioral teleportation paradigms^125,126^.

The introduction of robotic fish into experimental settings has prompted several key research questions. One major inquiry focuses on whether fish treat robots as conspecifics and under what conditions they integrate them into their social networks. Studies have demonstrated that fish exhibit preferences for robotic conspecifics that closely mimic natural swimming patterns and body shapes. For instance, golden shiners (*Notemigonus crysoleucas*) and guppies (*Poecilia reticulata*) showed a higher acceptance of robotic fish with realistic eyes and natural motion patterns^127,128^. It was further demonstrated that biomimetic locomotion was critical for attracting live fish to swim alongside a robotic fish, emphasizing that hydrodynamics and propulsion methods must closely match those of real fish to ensure effective integration^129^. Similarly, zebrafish show a preference for biomimetic robots that replicate species-specific color patterns and movement dynamics, reinforcing the idea that visual and motion cues play a significant role in social attraction and group cohesion^130,131^.

Another central question is whether robotic fish can simulate different social roles within a shoal. For instance, Faria and colleagues^112^ introduced a biomimetic robot designed to interact with three-spined sticklebacks. Their experiments revealed that fish followed the robot fish as they would a real leader, demonstrating that robots can effectively influence collective decision-making. Moreover, when the robots adjusted their behavior based on real-time feedback from live fish, this resulted in higher leadership success^132^. This aligns with findings by Papaspyros and colleagues^122^, who showed that a robotic fish that alternated between following and leading behaviors was more readily accepted by a zebrafish shoal than one that imposed a strict leadership role. The ability to adapt behavior dynamically allows robotic fish to interact more naturally with their biological counterparts and mimic real fish-to-fish interactions.

Research has also examined how robotic fish can be used to study risk perception and predator avoidance. The response of newborn guppies to a robotic predator revealed that the presence of an artificial threat significantly altered schooling behavior, reinforcing the idea that robotic fish can be used to model ecological scenarios with high precision^133^. Along the same line of research, Abaid and colleagues^134^ found that golden shiners exhibit different risk-taking behaviors when exposed to robotic fish, suggesting that social robots can modulate fish responses to threats.

These studies suggest that robotic fish can serve as powerful experimental tools to manipulate and measure social dynamics in controlled environments and to investigate fundamental principles of behavioral coordination and plasticity, such as examining how individual fish adjust their social strategies based on environmental pressures.

Different types of robotic fish platforms have been developed, each with unique specifications tailored to different research needs. These platforms range from simple lure-based designs to highly sophisticated autonomous robots capable of real-time feedback control. Some robotic fish are designed for open-loop control, where they follow pre-programmed movement patterns without adapting to live fish responses^127,128,135–139^. These robots are useful for testing hypotheses about how fish react to fixed stimuli.

In contrast, closed-loop control robots adjust their behavior in real time based on the movements of live fish^113,119,140^. This interactive approach allows for more dynamic experiments, providing deeper insights into the feedback mechanisms underlying group coordination. Bonnet and colleagues^117,141^ designed a modular robotic system that combines a miniature wheeled mobile robot with a fish-like lure, enabling it to replicate small fish locomotion patterns with high fidelity. This platform was specifically developed to study direct underwater interactions with small fish species and to mimic their swimming behaviors. Another sophisticated platform, RoboTwin^120^, was introduced to study hydrodynamic interactions in schooling fish. By replicating real fish movement kinematics, RoboTwin allows researchers to measure power costs, thrust, and flow fields, providing insight into the energy-saving mechanisms of collective swimming. Swain and colleagues^142^, developed a real-time feedback-controlled robotic fish that integrates video processing and Bluetooth communication, enabling the robot to adjust its movements in response to live fish behavior. This innovation allows for interactive experiments that more closely mimic natural schooling dynamics.

Another category of robotic fish focuses on multi-agent systems, where multiple robotic fish interact with each other and with live fish to simulate complex shoaling behaviors. These platforms enable researchers to examine how fish respond to variations in group size, swimming synchronization, and social hierarchy^143^. Advances in material science have also led to the development of soft-bodied robotic fish, which use flexible materials to replicate the fluid motion of real fish more accurately, increasing their acceptance by live fish shoals^50,144^. New robotic platforms are also incorporating sensory feedback mechanisms that allow real-time adjustments in response to fish behaviors, such as adjusting speed, direction, or signaling patterns, enabling deeper insights into the role of sensory-motor integration in collective animal behavior^119^.

The findings from fish-robot interaction studies have provided significant insights into collective behavior. An important discovery is that fish can learn to predict robotic fish behavior over time. In guppies, it has been shown that fish can anticipate the movement of robotic fish over repeated interactions, indicating a learning process in response to artificial stimuli^139^. Additionally, experiments on social buffering have revealed that robotic fish can reduce stress in live fish, potentially offering applications for welfare improvements in aquaculture^138,145^. Research has also highlighted the role of sensory integration in fish-robot interactions, with studies showing that fish rely on both visual and hydrodynamic cues to assess the presence of conspecifics, further influencing their behavioral responses^114^. In weakly electric fish, biomimetic robotic dummies equipped with electric playback electrodes demonstrated that electrocommunication cues can override locomotor signals and are sufficient to recruit individuals and groups, highlighting the primacy of species-specific sensory channels in mediating social attraction and collective responses^100^. Such findings have implications for understanding the neural and sensory basis of social behavior, highlighting the importance of multimodal communication in maintaining coordinated group movement and reinforcing social bonds within aquatic communities. Moreover, the ability of robotic fish to evoke specific behavioral responses in different species has provided comparative insights into social cognition and the evolutionary basis of group dynamics, contributing to a broader understanding of collective intelligence in animal groups.

### Future research directions in investigating collective behavior

One of the primary future research directions will focus on the cognitive and behavioral processes that fish use to coordinate movement within a group. Traditional approaches rely on observational studies combined with computational analyses and modeling (see, for instance^146,147^, but robotic fish offer an unprecedented method to actively engage with real fish and test cognitive models in a controlled manner. A key question that robotic fish can help address is whether fish rely on global visual information to navigate or whether they selectively respond to a limited subset of influential neighbors. Some studies suggest that fish process a broad array of social cues^148,149^, while others indicate that they filter out most of their surroundings, prioritizing a few highly influential individuals^147,150,151^. Robotic fish, equipped with adaptive sensing and control systems, could be programmed to replicate various decision-making models and measure the real-time responses of live fish. Moreover, different fish species may exhibit distinct social processing strategies based on ecological factors such as group size, habitat complexity, and predation pressure. By varying robotic behavior across experimental conditions, one could identify whether general principles govern swimming coordination and social cognition across species or if species-specific behavioral and cognitive mechanisms are at play. This approach could lead to a more refined understanding of how social information is processed and acted upon in different ecological contexts.

Another major area where robotic fish will be instrumental is in the study of collective escape responses to predators. Fish schools display remarkable coordination in rapidly propagating escape waves across large groups, yet the underlying principles remain elusive^152^. By introducing local and controlled disturbances using robotic fish, researchers could systematically analyze how localized threats trigger group-wide reactions. One promising avenue is testing the hypothesis that fish schools operate near a critical state, where minor disturbances can cascade into large-scale coordinated movements^153–155^. Robotic fish could simulate different levels of predatory threats, such as sudden changes in speed, directional shifts, or vibrational cues, to quantify how individual responses scale up to collective behavior.

## Robots and fish: what next?

**Fig. 3:**
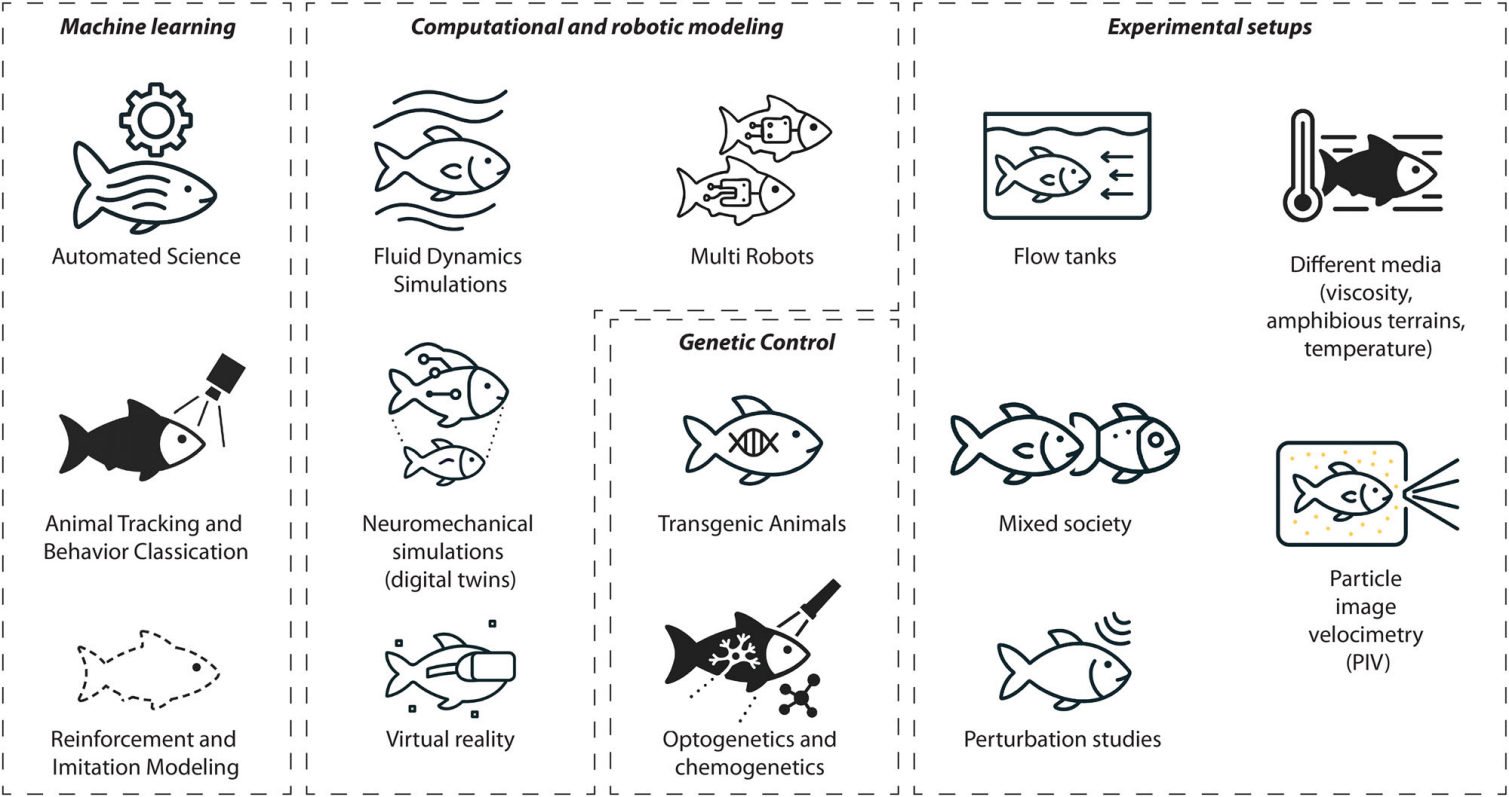
List of experimental tools available for future investigations of single and multiple fish swimming. Currently, most studies use only a small subset of these tools, and we expect that new combinations of them will allow exciting opportunities for progress in the field.

Recent technological advances bring many new opportunities both in terms of experimental setups and of scientific investigations (Fig. 3). In neuroscience, important progress is currently being made with the generation of transgenic animals, for instance zebrafish, and multiple options for short and long-term recording, stimulating or inhibiting particular neurons during behavior through opto- and chemo-genetics^156,157^.

As reviewed above, robotics and experimental setups are also progressing with increasingly better robots, and the availability of flow tanks equipped with particle image velocimetry (PIV) for flow characterization and virtual reality for stimulating fish and robots with arbitrary visual scenes^158^.

Simulation tools are also rapidly improving with faster and more accurate fluid simulators, GPU-based physics simulators, and neuromechanical simulation frameworks (simulation of environment, musculoskeletal system, and neural circuits). In the future, it might be possible to run imitation pipelines, in which videos of fish swimming are processed in real-time to create digital twins that would provide estimation of internal states (e.g., muscle contractions and sensory signals) and allow for making state-dependent perturbations (e.g., applying perturbations to the real fish that depend on the estimated internal states).

Machine learning is also helping on multiple fronts with marker-less animal tracking and behavior classification, optimization of robot controllers with deep reinforcement learning, and imitation learning^159^. Reinforcement learning is useful for exploring potential behaviors, and for instantiating parameters (e.g., synaptic weights or muscle parameters) in robot controllers or neuromechanical simulations given high-level target criteria (e.g., speed or energy efficiency). Similarly, imitation learning is useful for finding parameters that can produce a specific swimming gait (kinematic matching). Machine learning can even contribute to automated science, namely the use of information theory and optimization to propose new hypotheses and iteratively run experiments that optimize the collection of new knowledge in an automated way^160^, as well as new approaches for extracting analytical laws from data^161^.

So far, most studies have combined only a small subset of the possible experimental techniques described in Fig. 3, and new experimental techniques will be developed with time. In the future, we expect studies that will combine multiple of those together to address the open questions we discussed above. For instance, sensorimotor behaviors that involve multiple sensor modalities, such as rheotaxis, obstacle avoidance, and prey avoidance, could be studied in robots and animals with experimental set-ups that combine flow tanks, PIV, virtual reality, optogenetics, and perturbation studies (for instance, random pushes with water jets). This could be extended to schooling with several robots that play different roles: conspecific, prey or predator.

While laboratory studies offer controlled conditions, the future of robotic fish research also lies in their deployment in natural aquatic environments. By integrating three-dimensional movement capabilities and adaptive behavior algorithms, robotic fish could blend into wild fish populations and provide real-time data on natural schooling dynamics. One key application is environmental monitoring. Robotic fish equipped with environmental sensors could track temperature, water quality, and pollutant levels while simultaneously analyzing how these factors impact collective fish behavior. Such research would be invaluable for understanding the ecological determinants of schooling patterns and for assessing the effects of climate change and habitat degradation. Additionally, robotic fish could be programmed to learn and adapt to specific species’ behavioral rules using deep learning algorithms^162^. This capability would allow researchers to study species whose social structures and interaction rules are currently unknown. Lastly, robotic fish could potentially influence and control the collective behavior of wild fish schools. This application could have conservation implications, such as guiding fish away from hazardous areas (e.g., oil spills or illegal fishing zones) or encouraging migration patterns beneficial for ecosystem stability.

Such deployments in natural aquatic environments will, however, require further technological developments in order to ensure safe and successful missions. Among others, it will be important to improve energy storage and harvesting, fault-tolerance, and underwater localization and navigation (without GPS). Furthermore, the environmental impact of robot attrition (i.e., the permanent loss or unrecoverable failure of a robot during deployment) should be carefully assessed. Ideally, the next generation of fish robots should follow the recent efforts of designing biodegradable robotic devices^163–165^.

Finally, to date, simulation and robotic models have been explored using common and tractable biological models. As mentioned above a few animal models, such as zebrafish, have become powerful tools because they are easy to keep, reproduce quickly and are robust to behavioral, genetic and physiological manipulation. As the robotics field grows and technologies advance, it is important to consider the immense taxonomic and ecological scope of biological diversity that exists and is yet to be understood. This biological diversity in fishes provides a vast landscape of evolutionary ‘experiments’ that can be explored through simulation and robotics. As biology learns more about unique species with specialized functions, models will provide insights into the mechanisms that allow these species to flourish.

An exciting future lays therefore ahead. By addressing all these questions and challenges, roboticists and scientists will certainly improve our understanding of fish, as well as produce new fish-like robots with multiple potential applications in environmental monitoring, wild life tracking, inspection of facilities (sewers, pipes, dams, tanks, offshore platforms, fish farms), as well as serving as aquatic drones for hobbyists and outdoor enthusiasts.
